# Prevalence of Panton-Valentine leukocidin in methicillin-resistant *Staphylococcus aureus* clinical isolates at a university hospital in Northern Cyprus: a pilot study

**DOI:** 10.1186/s13104-020-05339-0

**Published:** 2020-10-20

**Authors:** Danyar Hameed M. Amin, Emrah Guler, Buket Baddal

**Affiliations:** 1 Department of Medical Microbiology and Clinical Microbiology, Faculty of Medicine, Near East University, Near East Boulevard, 99138 Nicosia, Cyprus; 2 Microbial Pathogenesis Research Group, DESAM Institute, Near East University, 99138 Nicosia, Cyprus

**Keywords:** Methicillin-resistant *Staphylococcus aureus*, Panton-Valentine leukocidin, PCR, Virulence, Prevalence, Northern Cyprus

## Abstract

**Objective:**

Panton-Valentine leukocidin (PVL)-positive methicillin-resistant *Staphylococcus aureus* (MRSA) is a healthcare problem worldwide. There are no reports on the virulence characteristics of MRSA in Northern Cyprus (NC). This study aimed to determine the presence of *pvl* among MRSA isolates from patients admitted to a university hospital in NC using molecular methods. Fifty *S. aureus* strains were included in this study. BD Phoenix automated identification system was used for bacterial identification and antibiotic susceptibility testing. Methicillin resistance was confirmed by disc diffusion assay. Presence of *nuc* and *mecA* genes was tested by multiplex PCR. Detection of *pvl* gene was performed by single-target PCR.

**Results:**

Out of 50 *S. aureus* isolates identified as MRSA by BD Phoenix system, 3 were susceptible to cefoxitin with disc diffusion assay and were confirmed as methicillin-sensitive *S. aureus* (MSSA). All isolates (n = 50, 100%) tested positive for the presence *nuc* gene and 68% (n = 34/50) were *mecA* positive. *pvl* was detected in 27.7% (n = 13/47) of the MRSA isolates. Among PVL-positive MRSA isolates, 69.2% (9/13) were inpatients. PVL-MRSA was more common in isolates from deep tracheal aspirate (30.8%, 4/13) and abscess/wound (23.1%, 3/13). This represents the first study of PVL presence among MRSA in hospital setting in NC.

## Introduction

*Staphylococcus aureus*, particularly methicillin-resistant *S. aureus* (MRSA), is a major human pathogen which causes both hospital- and community-acquired infections worldwide [1]. MRSA is commonly associated with skin and soft tissue infections (SSTIs), as well as pneumonia, bacteremia, and sepsis [2]. β-lactam antibiotic resistance in MRSA is attributed to the acquisition of *mecA* gene encoding the transpeptidase penicillin-binding protein 2a [3], and is a molecular hallmark for MRSA strains. The *mecA* gene is located on a mobile genetic element, the staphylococcal cassette chromosome *mec* (SCC*mec*), with at least 13 different types of SCC*mec* reported to date [4]. More recently, new *mecA* homologues *mecB*, *mecC* and *mecD* have also been identified to confer resistance to β-lactam antibiotics [5–7]. MRSA infections have been traditionally classified as either community-associated (CA-MRSA) or healthcare-associated MRSA (HA-MRSA). From a molecular standpoint, the CA-MRSA and HA-MRSA classifications are no longer distinct as patients can be colonized by MRSA in one setting and develop manifestations of infection in another. HA- and CA-MRSA infections have been conveniently used as epidemiological terms, however the line between them is significantly blurred. Although there are established healthcare-associated risk factors for HA-MRSA infection including recent hospitalization or surgery, residence in a long-term–care facility, dialysis, and indwelling percutaneous medical devices and catheters, community-onset HA-MRSA infections have been observed with increasing frequency among patients in community settings. Similarly, highly successful community-based clones have invaded the healthcare setting and have become successful nosocomial pathogens [8–11]. While demarcation of MRSA isolates as HA-MRSA or CA-MRSA can be confusing, there are clear differences in phenotypes and genetic background of MRSA strains associated with infection in either setting. HA-MRSA typically harbour SCC*mec* types I, II, or III, whereas CA-MRSA isolates carry SCC*mec* types IV, V and VI [12, 13].

Clinically, CA-MRSA is mainly associated with SSTIs [14] and often produce Panton-Valentine leukocidin (PVL), encoded by two co-transcribed genes, LukS-PV and LukF-PV [15, 16]. PVL is a bicomponent pore-forming cytotoxin assembled by LukS-PV and LukF-PV, has been demonstrated to have a significant role in the pathogenesis of MRSA by selectively targeting polymorphonuclear cells, macrophages and monocytes [17]. Epidemiological and clinical data provide substantial evidence that the high virulence potential of CA-MRSA isolates is associated with the expression of PVL [18, 19], which was also shown to be an important contributing factor in CA-MSSA infections, particularly in severe SSTIs and most notably in necrotizing pneumonia [14, 20, 21]. While HA-MRSA strains were initially observed not to be associated with PVL production [18], PVL genes carried by HA-MRSA strains have also been recently described in cutaneous and invasive infections [22, 23].

Evidently, the epidemiology of MRSA strains is constantly changing and their prevalence as well as molecular characteristics are known to vary between hospitals in different countries, cities within a country, or among wards of a hospital. Therefore, surveillance of the changing epidemiology of MRSA in local healthcare facilities with unique patient population is crucial for obtaining data that may aid empirical therapy and patient management. There are no reports on the molecular detection of virulence characteristics or their prevalence in MRSA isolates in Northern Cyprus (NC) in literature. This study aimed to investigate the prevalence of *pvl* in MRSA clinical isolates from patients admitted to a university hospital in NC using molecular methods.

## Main text

### Methods

#### Bacterial isolates

In this study, 50 *S. aureus* clinical isolates from patients admitted to Near East University Hospital between January 2012 and December 2019 which were processed at the hospital microbiology laboratory were randomly selected and included. Demographic data from each patient was anonymously collected and digitally stored. Bacterial isolates were initially cultured on blood agar media and were incubated at 37 °C for 18–24 h. All isolates were screened with coagulase test. Isolates were consequently processed with BD Phoenix 100 (Becton–Dickinson, BD Diagnostic Instrument Systems, USA) automated bacterial identification system according to supplier recommendations.

#### Antimicrobial susceptibility testing

Antimicrobial susceptibility of all isolates was initially performed by BD Phoenix 100 system. Consequently, susceptibility to cefoxitin in all *S. aureus* isolates identified as methicillin resistant with BD Phoenix 100 system was confirmed by agar disc diffusion assay and interpreted according to the European Committee on Antimicrobial Susceptibility Testing (EUCAST) guidelines [24]. Briefly, isolates were cultured on Mueller Hinton agar using 0.5 McFarland inoculum and cefoxitin disc (30 µg) was used for screening. All plates were incubated at 35 °C 5% CO_2_ for 24 h. Bacterial isolates with a zone of inhibition diameter < 22 mm were determined to be MRSA, while isolates with a zone of inhibition diameter ≥ 22 mm were recorded as MSSA.

#### Rapid DNA extraction method

DNA extraction was performed using the boiling method described previously [25]. Briefly, one to two colonies from overnight cultures on blood agar plates were suspended in 500 µl of sterile distilled water, and the suspension was heated at 100 °C for 15 min. After centrifugation for 5 min at 14,000 rpm to sediment the debris, 2 µl of the clear supernatant was used as template for PCR amplification.

#### Multiplex PCR detection of *mecA* and *nuc* genes

A multiplex PCR assay for the detection of *S. aureus* species-specific thermonuclease gene (*nuc*) and *mecA* gene was performed for all isolates. *S. aureus* SCC*mec* type IV strain (*nuc* +, *mecA* +, *pvl* −) was used as amplification control and pure water was used as negative control. Multiplex PCR assay included 2 µl of the DNA template added to a 25 µl final reaction mixture containing: 2X PCR Master Mix (Thermo Fischer Scientific, Waltham, MA USA) containing reaction buffer, Taq DNA polymerase (0.05 U/µL), 4 mM MgCl_2_, 0.4 mM of each dNTP and 10 pmol of each primer. The primer sets were used for amplification are shown in Additional file 1: Table S1 [26] and amplification was performed as described before [27] with a modification of an initial denaturation step at 94 °C for 10 min. PCR products were analyzed by agarose gel electrophoresis using 1.5% agarose gel. Gels were stained with ethidium bromide and visualized using MiniBIS Pro Gel Imaging System (DNR, Israel).

#### PCR detection of *pvl* gene

The presence of *pvl* gene was investigated for each sample using conventional single target PCR. 25 µl reaction mixture was prepared as described above using primer sets shown in Additional file 1: Table S1 [14]. *S. aureus* SCC*mec* type II strain (*nuc* +, *mecA* +, *pvl* +) was used as amplification control and pure water was used as negative control. PCR amplification was performed using conditions described before [28] with a modification of an initial denaturation step at 94 °C for 10 min. Product amplification was analyzed using gel electrophoresis.

### Results

**Fig. 1:**
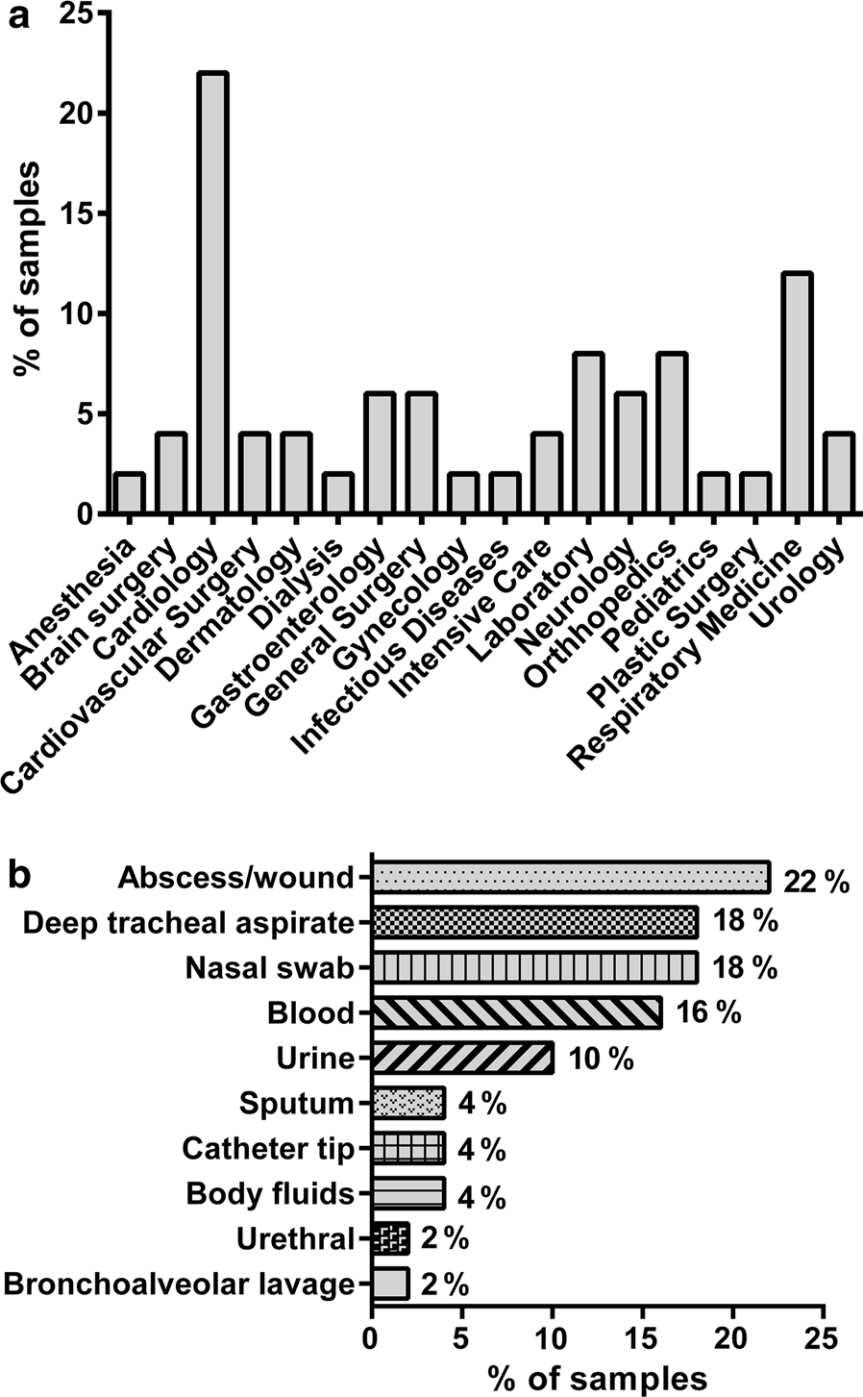
Distribution of *Staphylococcus aureus* infections **a** according to hospital departments **b** according to body sites

Fifty *S. aureus* strains isolated from different isolation sites from patients admitted to various hospital departments were investigated in this study. Within the patient group, 72% were inpatients (36/50) and 28% (14/50) were outpatients (Additional file 2: Figure S1). Of all the patients, 56% (28/50) were males and 44% (22/50) were females (Additional file 3: Figure S2). Clinical samples from patients were obtained from a range of departments were included in this study. The highest number of *S. aureus* cases were observed in Cardiology and Respiratory Medicine departments. Distribution of cases according to hospital departments is shown in Fig. 1a. Clinical samples were from collected from diverse patient body sites. Analysis of sample isolation sites indicated that 22% (n = 11) of the samples were taken from abscess/wound, 18% (n = 9) deep tracheal aspirate (DTA), 18% (n = 9) nasal swab, 16% (n = 8) of the samples were from blood, 10% (n = 5) urine, 4% (n = 2) sputum, 4% (n = 2) catheter tip, 4% (n = 2) body fluids, 2% (n = 1) bronchioalveolar lavage (BAL) and 2% (n = 1) were from urethral origin. Data suggested that the highest number of *S. aureus* cases were observed in samples isolated from abscess/wound, followed by DTA and nasal swabs. Analysis of different isolation sites for all samples is shown in Fig. 1b.

**Fig. 2:**
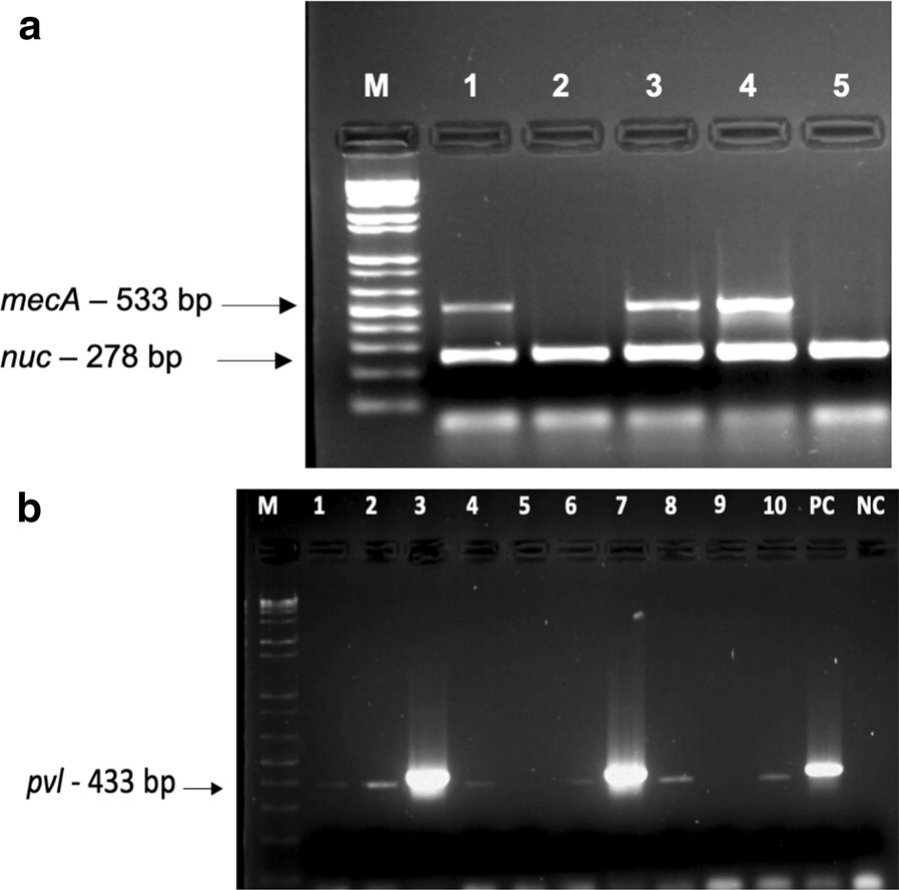
PCR detection of *nuc*, *mecA* and *pvl* genes **a** multiplex PCR for simultaneous detection of *nuc* and *mecA* in MRSA isolates. Lanes 1 and 3: *nuc, mecA* positive, Lane 2: *nuc* positive, PC: positive control, NC: negative control, M: 100 bp DNA ladder (Hibrigen); **b** detection of *pvl* in MRSA isolates by single target PCR. Lanes 1–4, 6–8, 10: *pvl* positive, Lanes 5, 9: *pvl* negative, PC: positive control, NC: negative control, M: 100 bp DNA ladder (Hibrigen)

Out of 50 *S. aureus* isolates, previously identified as methicillin-resistant by the BD Phoenix 100 automated bacterial identification system, 3 isolates were detected to be susceptible to methicillin based on cefoxitin disc diffusion assay and were confirmed as MSSA. Among the 50 isolates, 100% (50/50) were *nuc* positive by PCR. *mecA* gene was detected in 68% (34/50) of the isolates as confirmed with the multiplex PCR assay (Fig. 2a). Isolates which were detected as cefoxitin resistant by disc diffusion assay (n = 47) but did not carry *mecA* gene required further screening to identify alternative mechanisms of resistance by *mecB* and *mecC* PCR. Overall, 13 isolates out of 47 were positive for *pvl* gene, and the prevalence of *pvl* in confirmed MRSA isolates was 27.7% (13/47) (Fig. 2b). Two PVL-positive isolates which were cefoxitin susceptible by disc diffusion assay and negative for the presence of *mecA* were excluded from prevalence analysis. The clinical and molecular characteristics of the PVL-positive isolates are summarized in Table 1.

**Table 1:** Characteristics of Panton-Valentine leukocidin-positive isolates of *Staphylococcus aureus*

Patient age	Admission	Specimen	Ward	*nuc*	*mecA*	*pvl*
25	Outpatient	Urine	Gynecology	+	+	+
99	Inpatient	DTA	Respiratory medicine	+	+	+
66	Inpatient	Catheter tip	Cardiology	+	+	+
58	Inpatient	BAL	Respiratory medicine	+	+	+
34	Inpatient	Nasal swab	Cardiology	+	+	+
1	Outpatient	Urine	Pediatrics	+	+	+
63	Inpatient	Nasal swab	Cardiovascular surgery	+	+	+
41	Inpatient	DTA	Respiratory medicine	+	+	+
55	Outpatient	Abscess/wound	Dermatology	+	+	+
20	Outpatient	Abscess/wound	Dermatology	+	+	+
28	Inpatient	Abscess/wound	General surgery	+	+	+
96	Inpatient	DTA	ICU	+	+	+
79	Inpatient	DTA	ICU	+	+	+
37	Outpatient	Abscess/wound	General surgery	+	- *	+
55	Inpatient	Urine	Urology	+	- *	+

*ICU* intensive care unit, *DTA* deep tracheal aspirate, *BAL* bronchoalveolar lavage

*These isolates were excluded from *pvl* prevalence analysis

Among the PVL-positive MRSA isolates 69.2% (9/13) were inpatients. PVL positivity was more common in MRSA isolated from DTA (30.8%, 4/13) and abscess/wound (23.1%, 3/13). The PVL-positive isolates were from nine ward areas, but were mainly from the Respiratory Medicine, Cardiology and Dermatology departments, as well as the Intensive Care Unit (ICU).

### Discussion

Spread of PVL-producing MRSA from the community into healthcare setting poses a great public health risk and may result in outbreaks affecting vulnerable populations such as neonatal and intensive care units. Screening of virulence characteristics of patient-derived isolates is therefore essential for timely identification of patients carrying multi-drug resistant and virulent bacterial strains that would enable their isolation in hospitals. This pilot study reports novel findings in relation to MRSA virulence traits in a hospital setting in NC, in which the prevalence of PVL-positive MRSA was determined to be 27.7%.

Epidemiological characteristics of bacterial strains vary among countries, cities, hospitals as well as different wards within a hospital. So far, multiple studies have reported the presence of PVL in MRSA strains circulating in the hospital setting. In Turkey, PVL-positivity in MRSA isolates in hospitals was reported to range between 1.7 and 20% [29–32]. In a 2010 study in England and Wales, programme of enhanced surveillance of PVL-MRSA indicated an increased trend in clinical specimens [33], with reports of PVL-positive MRSA outbreak in a regional neonatal unit in the UK in 2012 [34]. Large-scale analysis of pediatric patients with invasive *S. aureus* infections recruited from 13 centers in 7 European countries has revealed that overall PVL positivity rate in MRSA isolates in 2016 was 7.8% [21]. On the other hand, reports show that the prevalence of *pvl* gene among MRSA strains in a Japanese hospital in 2019 was 13.5% [35], whereas PVL-positivity was higher at a rate of 39% among MRSA isolates collected from pediatric patients in China within the same year [36]. Epidemiological surveillance of MRSA strains collected between 2012 and 2017 in Australia showed that 28% of the isolates were PVL positive [37]. In an attempt to investigate MRSA isolates in developing countries, Shrestha et al. screened MRSA isolates at a tertiary hospital in Nepal, and indicated *pvl* gene positivity rate in nosocomial isolates to be 26.1% [38]. In another study, Singh-Moodley et al. have characterized MRSA isolates from blood cultures from 2013 to 2016 in South Africa, and reported *pvl* positivity rate to be 25% [39]. PVL-MRSA has been also linked to nosocomial infections in which hospital transmission of CA-MRSA had occurred in the US [40], highlighting the important source of CA-MRSA as a public health threat in hospitals. In low MRSA prevalence settings, healthcare workers may also serve as a reservoir of MRSA and an important potential source of transmission to patients as demonstrated by several studies [41, 42].

Several EU-EEA countries are still reporting high levels of MRSA. Between 2003 and 2005, Cyprus was reported to have highest proportion of MRSA among many countries in the Mediterranean region with a prevalence of 64%, while the prevalence of invasive MRSA cases between 2015 and 2018 were reported to range between 25 and < 50% [43], indicative of a high burden to the healthcare system. There is a shortage of data in terms of molecular characteristics of clinical *S. aureus* isolates in Cyprus. Our findings show, for the first time, that 27.7% of MRSA strains isolated from patients in a university hospital in NC were *pvl* positive. Continued surveillance and characterization of MRSA isolates in hospitals in the country is imperative for the prevention of spread of virulent nosocomial infections and the implementation of enhanced infection control strategies. Further in-depth molecular typing of clinical MRSA isolates should also be pursued to identify MRSA SCC*mec* types circulating both in the community and hospital setting.

## Limitations

This was a pilot study with a small sample size of 50 isolates from a single center, and therefore does not represent an overall prevalence of PVL-MRSA in hospitals in Cyprus. Studies with a larger number of samples collected from patients admitted to different hospitals across the country are needed to determine the overall prevalence and distribution of virulent strains. Another major limitation of the study was the lack of genotyping to demonstrate SCC*mec*, Multi-Locus Sequence Typing (MLST), protein A (*spa*) and accessory gene regulator (agr) types of isolates and further analysis of additional virulence factors associated with MRSA infection.

## Supplementary information

**Additional file 1: Table S1.** Specifications of designed primers.

**Additional file 2: Figure S1.** Admission type for patients with *S. aureus* infection.

**Additional file 3: Figure S2.** Gender of patients with *S. aureus* infection.

## Data Availability

The datasets used and/or analysed during the current study are available from the corresponding author on reasonable request.

## Abbreviations

PVL: Panton-Valentine leukocidin
NC: Northern Cyprus
PCR: Polymerase chain reaction
MRSA: Methicillin-resistant *Staphylococcus aureus*
SSTIs: Skin and soft tissue infections
SCC*mec*: Staphylococcal cassette chromosome *mec*
CA-MRSA: Community-associated MRSA
HA-MRSA: Healthcare-associated MRSA
EUCAST: European Committee on Antimicrobial Susceptibility Testing
DTA: Deep tracheal aspirate
BAL: Bronchoalveolar lavage
ICU: Intensive care unit

## References

[1] Stefani S, Goglio A (2010). Methicillin-resistant *Staphylococcus aureus*: related infections and antibiotic resistance. Int J Infect Dis..

[2] Chambers HF, DeLeo FR (2009). Waves of resistance: *Staphylococcus aureus* in the antibiotic era. Nat Rev Microbiol.

[3] Hartman BJ, Tomasz A (1984). Low-affinity penicillin-binding protein associated with β-lactam resistance in *Staphylococcus aureus*. J Bacteriol.

[4] Lakhundi S, Zhang K (2018). Methicillin-resistant *Staphylococcus aureus*: molecular characterization, evolution, and epidemiology. Clin Microbiol Rev.

[5] Becker K, van Alen S, Idelevich EA (2018). Plasmid-encoded transferable mecb-mediated methicillin resistance in *Staphylococcus aureus*. Emerg Infect Dis.

[6] Schwendener S, Cotting K, Perreten V (2017). Novel methicillin resistance gene mecD in clinical *Macrococcus caseolyticus* strains from bovine and canine sources. Sci Rep..

[7] García-Álvarez L, Holden MTG, Lindsay H (2011). Meticillin-resistant *Staphylococcus aureus* with a novel mecA homologue in human and bovine populations in the UK and Denmark: a descriptive study. Lancet Infect Dis..

[8] Uhlemann AC, Otto M, Lowy FD (2014). Evolution of community- and healthcare-associated methicillin-resistant *Staphylococcus aureus*. Infect Genet Evol..

[9] Seybold U, Kourbatova EV, Johnson JG (2006). Emergence of community-associated methicillin-resistant *Staphylococcus aureus* USA300 genotype as a major cause of health care-associated blood stream infections. Clin Infect Dis.

[10] Choo EJ (2017). Community-associated methicillin-resistant *Staphylococcus aureus* in nosocomial infections. Infect Chemother..

[11] Maree CL, Daum RS, Boyle-Vavra S (2007). Community-associated methicillin-resistant *Staphylococcus aureus* isolates causing healthcare-associated infections. Emerg Infect Dis.

[12] David MZ, Daum RS (2010). Community-associated methicillin-resistant *Staphylococcus aureus*: epidemiology and clinical consequences of an emerging epidemic. Clin Microbiol Rev.

[13] Kong EF, Johnson JK, Jabra-Rizk MA (2016). Community-associated Methicillin-resistant *Staphylococcus aureus*: an enemy amidst us. PLoS Pathog.

[14] Lina G, Piemont Y, Godail-Gamot F (1999). Involvement of Panton-Valentine Leukocidin-producing *Staphylococcus aureus* in primary skin infections and pneumonia. Clin Infect Dis.

[15] Vandenesch F, Naimi T, Enright MC (2003). Community-acquired methicillin-resistant *Staphylococcus aureus* carrying panton-valentine leukocidin genes: worldwide emergence. Emerg Infect Dis.

[16] Diep BA, Sensabaugh GF, Somboona NS (2004). Widespread Skin and Soft-Tissue Infections Due to Two Methicillin-Resistant *Staphylococcus aureus* Strains Harboring the Genes for Panton-Valentine Leucocidin. J Clin Microbiol.

[17] Yoong P, Torres VJ (2013). The effects of *Staphylococcus aureus* leukotoxins on the host: cell lysis and beyond. Curr Opin Microbiol.

[18] Gillet Y, Issartel B, Vanhems P (2002). Association between *Staphylococcus aureus* strains carrying gene for Panton-Valentine leukocidin and highly lethal necrotising pneumonia in young immunocompetent patients. Lancet.

[19] Chambers HF (2005). Community-associated MRSA - Resistance and virulence converge. N Engl J Med.

[20] Chiu YK, Lo WT, Wang CC (2012). Risk factors and molecular analysis of Panton-Valentine leukocidin-positive methicillin-susceptible *Staphylococcus aureus* colonization and infection in children. J Microbiol Immunol Infect.

[21] Gijón M, Bellusci M, Petraitiene B (2016). Factors associated with severity in invasive community-acquired *Staphylococcus aureus* infections in children: a prospective European multicentre study. Clin Microbiol Infect.

[22] Mariem BJJ, Ito T, Zhang M (2013). Molecular characterization of methicillin-resistant Panton-valentine leukocidin positive *Staphylococcus aureus* clones disseminating in Tunisian hospitals and in the community. BMC Microbiol.

[23] Song JH, Hsueh PR, Chung DR (2011). Spread of methicillin-resistant *Staphylococcus aureus* between the community and the hospitals in Asian countries: an ANSORP study. J Antimicrob Chemother.

[24] European Committee on Antimicrobial Susceptibility Testing. Available from: https://www.eucast.org/fileadmin/src/media/PDFs/EUCAST_files/Breakpoint_tables/v_10.0_Breakpoint_Tables.pdf.

[25] Pérez-Roth E, Claverie-Martín F, Villar J (2001). Multiplex PCR for simultaneous identification of *Staphylococcus aureus* and detection of methicillin and mupirocin resistance. J Clin Microbiol.

[26] Merlino J (2002). Detection and expression of methicillin/oxacillin resistance in multidrug-resistant and non-multidrug-resistant *Staphylococcus aureus* in Central Sydney, Australia. J Antimicrob Chemother..

[27] Rahman MM, Amin KB, Rahman SMM (2018). Investigation of methicillin-resistant *Staphylococcus aureus* among clinical isolates from humans and animals by culture methods and multiplex PCR. BMC Vet Res..

[28] Japoni-Nejad A, Rezazadeh M, Kazemian H (2013). Molecular characterization of the first community-acquired methicillin-resistant *Staphylococcus aureus* strains from Central Iran. Int J Infect Dis..

[29] Akočlu H, Zarakolu P, Altun B (2010). Epidemiological and molecular characteristics of hospital-acquired methicillin-resistant *Staphylococcus aureus* strains isolated in Hacettepe University Adult Hospital in 2004–2005.

[30] Oksuz L, Dupieux C, Tristan A (2013). The high diversity of MRSA clones detected in a university hospital in Istanbul. Int J Med Sci..

[31] Kılıç A, Doğan E, Kaya S (2015). Investigation of the presence of mecC and Panton-Valentine Leukocidin genes in *Staphylococcus aureus* strains isolated from clinical specimens during seven years period. Mikrobiyol Bul..

[32] Duman Y, Sevimli R (2018). Investigation of the presence of pantone-valentine leukocidin in *Staphylococcus aureus* strains isolated from orthopedic surgical site infections. Mikrobiyol Bul..

[33] Ellington MJ, Ganner M, Smith IM (2010). Panton-Valentine Leucocidin-related disease in England and Wales. Clin Microbiol Infect.

[34] Ali H, Nash JQ, Kearns AM (2012). Outbreak of a South West Pacific clone Panton-Valentine leucocidin-positive meticillin-resistant *Staphylococcus aureus* infection in a UK neonatal intensive care unit. J Hosp Infect.

[35] Funaki T, Yasuhara T, Kugawa S (2019). SCCmec typing of PVL-positive community-acquired *Staphylococcus aureus* (CA-MRSA) at a Japanese hospital. Heliyon..

[36] Wang X, Shen Y, Huang W (2019). Characterisation of community-acquired *Staphylococcus aureus* causing skin and soft tissue infections in a children’s hospital in Shanghai, China. Epidemiol Infect..

[37] Dotel R, Sullivan MVN, Davis JS (2019). Molecular epidemiology of methicillin-resistant Staphylococcus aureus isolates in New South Wales, Australia, 2012–2017. Infect Dis Heal..

[38] Shrestha B, Singh W, Raj VS (2014). High Prevalence of Panton-Valentine leukocidin (PVL) genes in nosocomial-acquired *staphylococcus aureus* isolated from tertiary care hospitals in Nepal.

[39] Singh-Moodley A, Strasheim W, Mogokotleng R (2019). Unconventional SCCmec types and low prevalence of the Panton-Valentine Leukocidin exotoxin in South African blood culture *staphylococcus aureus* surveillance isolates, 2013–2016. PLoS ONE.

[40] Saiman L, Keefe MO, Graham PL (2003). Hospital transmission of community-acquired Methicillin-resistant *staphylococcus aureus* among postpartum women. Clin Infect Dis.

[41] Lari AR, Pourmand MR, Ohadian Moghadam S (2011). Prevalence of PVL-containing MRSA isolates among hospital staff nasal carriers. Lab Med..

[42] Papastergiou P, Tsiouli E (2018). Healthcare-associated transmission of Panton-Valentine leucocidin positive methicillin-resistant *staphylococcus aureus*: the value of screening asymptomatic healthcare workers. BMC Infect Dis.

[43] ECDC Surveillance Atlas - Antimicrobial resistance. https://www.ecdc.europa.eu/en/antimicrobial-resistance/surveillance-and-disease-data/data-ecdc.

