# Supplementary material for: Prevalence of Panton-Valentine leukocidin in methicillin-resistant Staphylococcus aureus clinical isolates at a university hospital in Northern Cyprus: a pilot study
Source: BMC Res Notes. 2020 Oct 20;13:490. doi: 10.1186/s13104-020-05339-0 (PMC7576721; doi:10.1186/s13104-020-05339-0)
Supplement: Supplementary file 1 — Additional file 1: Table S1. Specifications of designed primers. [file 13104_2020_5339_MOESM1_ESM.docx]

**Table S1** Specifications of designed primers

| Gene | Primer sequence (5′ to 3′) | Product size (bp) |
| --- | --- | --- |
| mecA-F | AAAATCGATGGTAAAGGTTGGC | 533 |
| mecA-R | AGTTCTGCAGTACCGGATTTGC |  |
| nuc-F | GCGATTGATGGTGATACGGTT | 278 |
| nuc-R | AGCCAAGCCTTGACGAACTAAAGC |  |
| Luk PV-F | ATCATTAGGTAAAATGTCTGGACATGATCCA | 433 |
| Luk PV-R | GCATCAAGTGTATTGGATAGCAAAAGC |  |
